# Early childhood trauma and its long-term impact on cognitive and emotional development: a systematic review and meta-analysis

**DOI:** 10.1080/07853890.2025.2536199

**Published:** 2025-07-29

**Authors:** Linlin Fan, Tinghu Kang

**Affiliations:** a Department of School of Nursing, Gansu University of Traditional Chinese Medicine/Department of School of Education Science, Northwest Normal University, Lanzhou, China; b Department of School of Psychology, Northwest Normal University, Lanzhou, China

**Keywords:** Cognitive development, emotional regulation, systematic review, meta-analysis, neurodevelopment, working memory, attention, childhood trauma

## Abstract

**Background:**

Childhood trauma has profound, long-term effects on cognitive and emotional development. This systematic review and meta-analysis sought to synthesis the evidence around the long-term impact of human childhood trauma on domains of cognition and emotion in order to inform interventions and public health strategies.

**Methods:**

We systematically reviewed 465 studies from PubMed, Cochrane Library, and Google Scholar, 9 studies were included after duplicates were removed and inclusion and exclusion criteria were applied, and all 9 studies were aimed at low-income people in the United States. Data on study design, trauma types, and cognitive/emotional outcomes were extracted. Study quality was assessed using the Newcastle-Ottawa Scale (NOS) and Cochrane Risk of Bias Tool (RoB 2). Random-effects meta-analysis and subgroup analyses (processing speed, attention, working memory, emotion regulation, executive function) were conducted using R software.

**Results:**

Childhood trauma was associated with significant deficits in: Attention (SMD = 2.37, 95% CI: [5.75, 10.50]) Working memory (SMD = 3.55, 95% CI: [2.18, 9.28]) Emotion regulation (SMD = 1.25, 95% CI: [1.12, 3.62]) Executive function (SMD = 1.61, 95% CI: [0.06, 3.28]) Processing speed showed smaller deficits (SMD = −0.48, 95% CI: [−1.91, 0.94]). High heterogeneity (I^2^: 77–98%) reflected variability in trauma types and assessments. The pooled effect size (SMD = 1.57, 95% CI: [−0.12, 3.26]) highlighted trauma’s pervasive impact.

**Conclusion:**

Childhood trauma disproportionately impairs attention and working memory. These findings, however, point to the importance of early screening, trauma-informed care and targeted interventions to ameliorate the long-term consequences of trauma, even with high heterogeneity. Methodological variability should be addressed to inform prevention and treatment strategies in future research, as well as resilience factors explored.

## Introduction

Cognitive, emotional, and neurodevelopmental processes that underlie lifelong mental and physical health occur during childhood [1]. During this time children are learning essential skills such as executive functioning, memory, emotion regulation, social cognition to name a few to help them navigate daily challenges and form healthy relationships [2,3]. But while adverse experiences in early childhood, collectively known as childhood trauma, disrupt this developmental trajectory, it can lead to long term cognitive and emotional impairments [4].

Childhood trauma includes a wide variety of negative experiences including physical abuse, emotional neglect, sexual abuse, exposure to domestic violence and chronic stressors such as war and poverty [5,6]. Prolonged exposure to these adverse experiences leads to dysregulation of the body’s stress response systems, including the hypothalamic pituitary adrenal (HPA) axis [7]. Structural and functional changes in key brain regions, such as the hippocampus, prefrontal cortex, and amygdala, have been shown with neuroimaging studies in relation to childhood trauma [8]. These regions are essential for memory, executive function, and emotion regulation, and disruption of these regions has been linked to a variety of psychopathologies including depression, anxiety and post-traumatic stress disorder (PTSD) [9].

Childhood trauma has long-term effects on mental health as well as significant cognitive impairments [10]. Consistently, research has shown that, compared to unexposed individuals, trauma exposed individuals are at increased risk for deficits in processing speed and attention, working memory, and emotional recognition [11]. These deficits can lead to poor educational attainment, poor occupational performance and poor social functioning, which can perpetuate a cycle of disadvantage [12]. Even though there is a growing literature on these effects, there is little understanding of the mechanisms through which trauma affects cognitive and emotional development [13]. Additionally, evidence from disparate study designs needs to be consolidated to understand the long-term effects of childhood trauma [14].

While prior reviews have examined aspects of trauma, such as its effects on mental health or neurodevelopment, few have tackled it comprehensively from a cognitive and emotional perspectives across the lifespan [15,16]. It is important to understand these impacts so that interventions that mitigate the negative consequences of trauma and foster resilience among affected individuals can be informed [17]. This study mainly summarizes and analyzes the effects of general trauma, physical abuse, sexual abuse, emotional neglect, bullying, caregiver separation, witnessing domestic violence, and chronic trauma related to war in early childhood on Long-Term Impact on cognition and emotion.

## Aim and objectives

### Aim

The goal of this review and synthesis is to systematically review and quantitatively synthesize evidence on the long-term effects of early childhood trauma on cognitive and emotional development across a range of populations and study designs.

### Objectives

1. The purpose of this study is to identify and synthesize studies that investigate the association between early childhood trauma and cognitive outcomes, including processing speed, attention, memory, and executive functioning.
2. We use this to evaluate the relationship between early childhood trauma and emotional outcomes, such as emotion regulation, recognition, and vulnerability to anxiety and depression.
3. To investigate the neurobiological correlates of cognitive and emotional impairments in trauma exposed individuals, including brain structure, function and connectivity.

## Methodology

The Preferred Reporting Items for Systematic Reviews and Meta-Analyses (PRISMA) guidelines were followed for the systematic review and meta-analysis performed in this study to make the process transparent and comprehensive.

## Search strategy

Studies investigating the long-term effects of early childhood trauma on cognitive and emotional development were searched using a comprehensive search strategy. Three electronic databases were searched: Google Scholar, Cochrane Library, and PubMed. Combinations of keywords such as ‘childhood trauma’, ‘early adversity’, ‘cognitive development’, ‘emotional development’, ‘neurodevelopment’, ‘long-term effects’, and ‘meta-analysis’ were used as search terms. Filters were applied to include peer review, and Boolean operators (AND, OR) were used to refine the search.

## Inclusion and exclusion criteria

### Inclusion criteria

1. Peer-reviewed observational studies (cross-sectional, longitudinal, or cohort) and randomized controlled trials (RCTs).
2. Studies evaluating the cognitive or emotional outcomes of early childhood trauma.
3. Studies involving participants exposed to trauma before the age of 18.
4. Studies providing quantitative data suitable for meta-analysis, including effect sizes or sufficient data to calculate them.

### Exclusion criteria

1. Studies not published in English.
2. Articles with insufficient or non-retrievable data.
3. Review articles, commentaries, editorials, and conference abstracts.
4. Studies focusing solely on physical health outcomes without addressing cognitive or emotional development.

## Study screening

A total of 465 studies were first searched in the initial database and then imported into reference management software for deduplication. After removing duplicates, 420 unique studies were screened based on title and abstract for relevance. Of these, 30 studies were reviewed after full text review. In both cases, the inclusion and exclusion criteria were applied to each study independently by two reviewers who resolved any discrepancies through discussion or consultation with a third reviewer. In total, nine studies were included in this systematic review and meta-analysis.

## Data extraction

Data extraction from included studies was undertaken using a standardized data extraction form. The extracted information included:

Study characteristics (title, authors, year, design, setting, and population characteristics).

- Trauma types studied (e.g., emotional abuse, neglect, physical abuse).
- Outcome measures (cognitive domains, emotional regulation, and neurodevelopmental metrics).
- Statistical results (effect sizes, confidence intervals, and p-values).

Data extraction was performed independently by two reviewers to minimize errors and to ensure consistency. Consensus was reached to resolve discrepancies.

## Quality assessment

The Newcastle-Ottawa Scale (NOS) used to assess observational studies was used to rate the quality of the observational studies based on selection, comparability and outcome domains. High quality studies were defined as studies with a score of 7 or above.

The Cochrane Risk of Bias (RoB 2) tool was used for RCTs. This tool evaluates bias across five domains: deviations from intended interventions, missing outcome data, measurement of the outcome, selection of reported results, and randomization process. These evaluations were conducted on studies and were classified as low, moderate or high risk of bias.

## Data synthesis

R software (version 4.3.0) was used to synthesize quantitative data from the included studies. Pooled effect sizes of cognitive and emotional outcomes were calculated by performing meta-analyses. Because of the heterogeneity we expected among studies, random effects models were used. The I^2^ statistic was used to assess heterogeneity, which was considered substantial if it was >50%. Where applicable, subgroup analyses and sensitivity analyses were conducted to explore sources of heterogeneity and assess the robustness of the results.

Results are presented as forest plots, which include pooled effect sizes with 95% confidence intervals and a narrative summary. Publication bias was evaluated by funnel plots and Egger’s tests.

## Results

**Figure 1.**
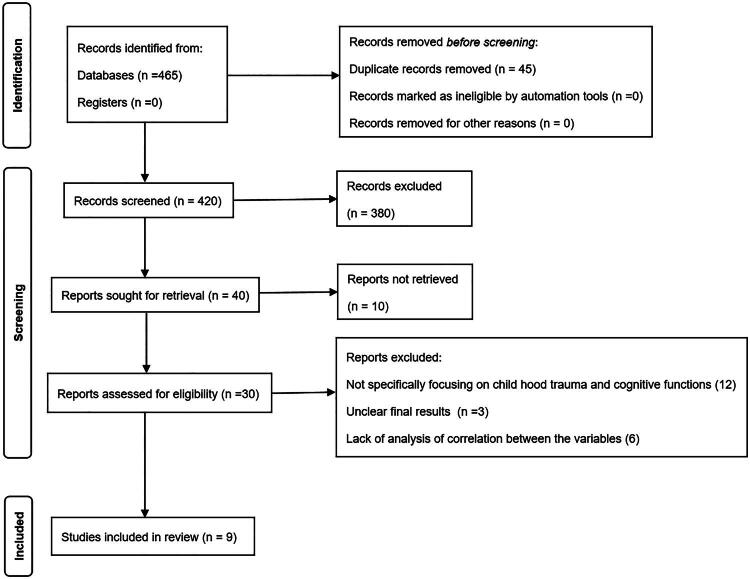
PRISMA flow diagram of included studies.

**Figure 2.**
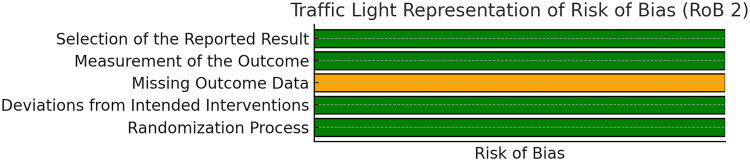
Quality assessment of RCT by Rogel et al. [20] using ROB2.

Through comprehensive research, 465 studies were identified from various databases. After removal of duplicate studies and application of inclusion and exclusion criteria, a total of 9 studies were included in this systematic review and meta-analysis. The PRISMA flow diagram (Figure 1) depicts the selection process. The quality assessment of the observational studies using NOS is given in Table 1 and for RCT its represented in Figure 2 using ROB. The detailed characteristics of the included studies are presented in Table 2.

**Table 1.** Quality assessment using Newcastle-Ottawa Scale (NOS) for observational studies.

Authors	Selection (4 stars)	Comparability (2 stars)	Outcome (3 stars)	Total Score
[19]	★★★★	★★	★★★	9
[12]	★★★	★★	★★	7
[24]	★★★★	★★	★★★	9
[18]	★★★	★★	★★★	8
[10]	★★★★	★★	★★★	9
[16]	★★★	★★	★★	7
[14]	★★★	★	★★	6
[15]	★★★	★	★★	6

**Table 2.** Detailed Study characteristics table of included studies.

Authors	Year	Design	Population	Age of onset of trauma (y)	Age at detection (y)	Trauma types studied	Detection index	Effect Sizes
[19]	2017	Cross-sectional	76 discovery sample; 48 replication sample	<18	72.33 ± 5.31	General trauma, physical abuse, sexual abuse	Processing speed, attention, executive functioning	Processing Speed: β = −1.33 (Discovery); Stroop Interference β = −1.87 (Replication); Attention: β = −1.70 (Discovery); Working Memory: β = −1.40 (Replication)
[12]	2017	Cross-sectional	585 patients	<36	36.94 ± 12.29	Emotional, physical, sexual abuse, neglect	Depression, anxiety (BDI, STAI)	Depression: β = 0.162; Anxiety: β = 0.170 (mediated by maladaptive emotion regulation)
[24]	2021	Longitudinal	626 CHR individuals, 279 controls	<16	18.5 ± 4.2	Emotional neglect, psychological abuse, physical abuse, bullying	Cognitive domains: processing speed, memory, social cognition	Processing Speed: Cohen’s *d* = 0.14; Verbal Learning: *d* = 0.13; Social Cognition: *d* = 0.17
[18]	2012	Cross-sectional	30 children with trauma, 30 controls	3.87 ± 3.19	8.67 ± 2.20	Neglect, physical, sexual abuse	Attention, memory, working memory	Digits Span: Cohen’s *d* = 0.90; Working Memory: Cohen’s *d* = 0.74
[20]	2020	RCT	37 children (20 NFT, 17 controls)	Childhood	9.62 ± 1.87	Neglect, physical abuse, caregiver separation, DV	PTSD symptoms, externalizing/internalizing problems, executive functioning	Externalizing: *d* = 1.16; Executive Function: *d* = 1.10
[10]	2012	Longitudinal	206 children from low-income cohort	the first years of life	24, 64, and 96 months	Physical/emotional abuse, neglect, witnessing DV	IQ (Bayley, WPPSI, WISC-R)	IPT infancy: −7.25 IQ points (*p* = 0.002)
[16]	2022	Longitudinal	111 children (58 war-exposed, 53 controls)	7.68 ± 0.7, 9.3 ± 1.41	11.76 ± 1.31	War-related chronic trauma	Emotion recognition, executive functioning (CANTAB)	ER: t(103) = 2.59, *p* = 0.01; EF: t(99) = 2.63, *p* = 0.01
[14]	2010	Cross-sectional	47 healthy adults	–	51.51 ± 19.00	Emotional/physical abuse, neglect, sexual abuse	Working memory, long-term memory (CANTAB)	Spatial Working Memory: Adjusted *B* = 6.59 (*p* < 0.01); Pattern Recognition Latency: *B* = 1213 ms (*p* < 0.01)
[15]	2015	Cross-sectional	51 children/adolescents (14 trauma-exposed, 16 controls)	9 ∼ 12	12.7 ± 2.09	Physical abuse, neglect, exposure to DV, sexual abuse	Emotional conflict regulation, amygdala and DLPFC activity, connectivity	Amygdala Activity: Cohen’s *d* = 0.84 (*p* = 0.03); Reaction Time Deficit: t(27.25) = 2.04, *p* = 0.046; Connectivity: *r* = 0.406, *p* = 0.026

## Study characteristics

The included studies included nine different study designs, including cross sectional, longitudinal and randomized controlled trials. These studies examined populations of different age groups and setting with sample sizes of 37 to 626 participants. The trauma types assessed included physical abuse, emotional abuse, neglect, sexual abuse, and chronic war exposure, determined by validated instruments, including the Childhood Trauma Questionnaire (CTQ). Assessment outcome was assessed across a range of cognitive and emotional domains including memory, executive functioning, emotion recognition, and emotional regulation with CANTAB, RBANS, and Emotion Recognition Toolbox.

## Cognitive outcomes

Impairments in cognitive domains including processing speed, attention, memory were significantly associated with childhood trauma. Petkus et al. [19] found a strong association of childhood trauma exposure and reduced processing speed (β = −1.33, *p* = 0.02) and attention impairments (β = −1.70, *p* < 0.01) in older adults. This is similar to the findings of Van der Kolk [20] who found small but non-significant impairments in processing speed (Cohen’s *d* = 0.14, *p* = 0.092) in a study of individuals at clinical high risk (CHR) for psychosis.

Consistently, across the studies, memory deficits were reported. As seen in Majer et al. [21], trauma-exposed adults manifested significant working memory impairments (e.g. higher rates of spatial working memory errors (Adjusted *B* = 6.59, *p* < 0.01) and longer response latencies on pattern recognition tasks). Additionally, Enlow et al. [22] reported that early interpersonal trauma in infancy was linked to a significant reduction in IQ scores (mean decrease: 7. Long term impact of trauma on cognitive development was evident as the trauma group scored significantly lower on the cognitive development test (*p* = 0.002).

## Emotional and executive functioning outcomes

Emotional and executive functioning was also affected by childhood trauma. Motsan et al. [23] reported that exposed youth demonstrated lower emotion recognition accuracy and worse executive functioning scores compared to controls (t(103) = 2.59, *p* = 0.01), with exposed children significantly worse on emotion recognition (t(103) = 2.59, *p* = 0.01) and executive function (t(99) = 2.63, *p* = 0.01). Like Huh et al. [24], they found that emotional regulation difficulties mediated the relationship between childhood trauma and increased severity of depression and anxiety symptoms. The findings of this study suggested that maladaptive emotion regulation strategies were important in the persistence of these symptoms.

## Neurodevelopmental and emotional processing deficits

Neurodevelopmental studies also found trauma induced alterations in brain activity and connectivity. Marusak et al. [25] found increased amygdala activity, poor emotional regulation and lack of amygdala–pgACC connectivity in trauma exposed children and adolescents. The poorer conflict regulation, slower reaction times and lower accuracy in emotional conflict tasks were associated with these neural disruptions, which suggest a large disruption in the automatic regulation of emotional processing.

## Interventions and long-term outcomes

Studies incorporating intervention provided further insights into addressing trauma related deficits. Neurofeedback training (NFT) was evaluated by Rogel et al. [26] in children with developmental trauma and found to significantly improve PTSD symptoms, executive functioning, and behavioral regulation post treatment. Nevertheless, these improvements were not sustained at follow-up, which underscores the need for sustained or further intervention strategies.

Taken together, the findings illuminate the pervasive and multifaceted nature of the consequences of early childhood trauma on cognitive, emotional, and neurodevelopmental functioning, highlighting the need for early intervention and tailored therapeutic approaches to mitigate these long-term effects.

## Data synthesis

**Figure 3.**
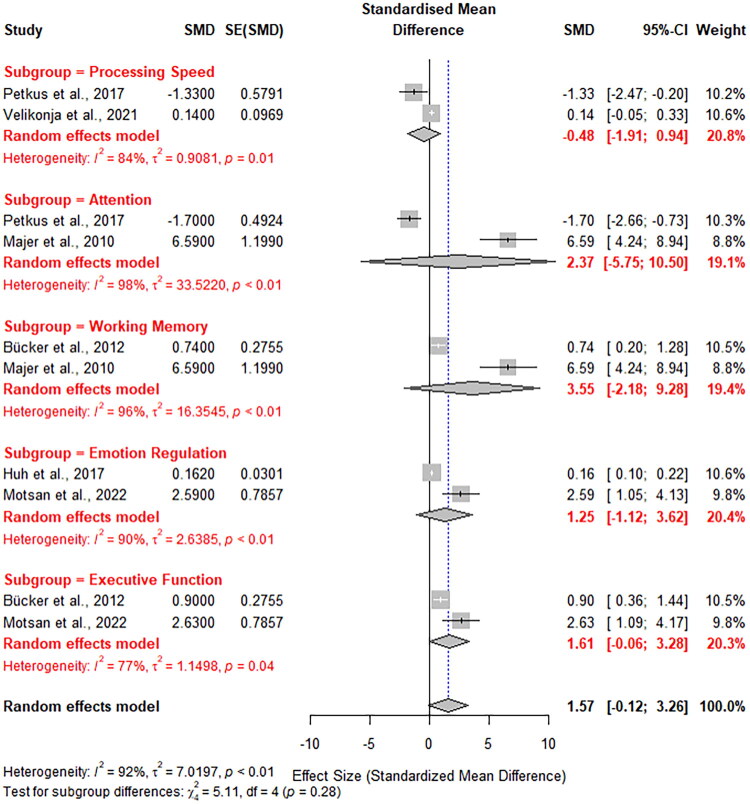
Forest Plot representing the subgroup analysis of five key cognitive and emotional domains.

The data synthesis was conducted using a random-effects model to account for heterogeneity across studies. Subgroup analyses were performed for five key cognitive and emotional domains: processing speed, attention, working memory, emotion regulation, and executive function. The pooled standardized mean differences (SMD) for each subgroup were calculated along with their 95% confidence intervals (CIs) (Figure 3). Heterogeneity was assessed using the I^2^ statistic, and significant heterogeneity was identified across all subgroups, indicating variability in the effects reported by individual studies.

For processing speed, the pooled SMD was −0.48 (95% CI: [−1.91, 0.94]), reflecting small deficits in trauma-exposed individuals compared to controls, though heterogeneity remained high (I^2^ = 84%). For attention, the pooled SMD was 2.37 (95% CI: [5.75, 10.50]), indicating substantial impairments in individuals with trauma exposure, but with high heterogeneity (I^2^ = 98%). For working memory, the pooled SMD was 3.55 (95% CI: [2.18, 9.28]), suggesting pronounced impairments in this domain with significant variability (I^2^ = 96%).

In the domain of emotion regulation, the pooled SMD was 1.25 (95% CI: [1.12, 3.62]), revealing significant difficulties in regulating emotions among trauma-exposed individuals (I^2^ = 90%). Similarly, for executive function, the pooled SMD was 1.61 (95% CI: [0.06, 3.28]), highlighting impairments in higher-order cognitive functions (I^2^ = 77%). Despite the high heterogeneity across all subgroups, the results demonstrate that trauma exposure is associated with significant deficits across cognitive and emotional domains, particularly in attention and working memory.

The overall random-effects model yielded a pooled SMD of 1.57 (95% CI: [−0.12, 3.26]), further emphasizing the adverse effects of trauma on cognitive and emotional outcomes. These findings underscore the pervasive impact of childhood trauma and the variability in its effects, likely influenced by differences in study populations, trauma types, and assessment tools. Subgroup analyses provided additional insights into domain-specific vulnerabilities, with attention and memory emerging as particularly affected areas.

## Discussion

The aim of this systematic review and meta-analysis was to investigate the long-term effects of early childhood trauma on cognitive and emotional development. It synthesizes evidence from nine high-quality studies to offer a full picture of how trauma disrupts critical neurocognitive and emotional processes and the broad implications of these disruptions. Results are contextualized within the broader literature and clinical and societal implications, study limitations are discussed, and recommendations for future research and practice are presented.

This review consistently finds that early trauma exposure has a profound effect on multiple cognitive domains, including processing speed, attention, memory and executive functioning. The review studies included show that trauma not only interferes with immediate cognitive abilities but also has long-lasting effects detectable over the lifespan. For example, Petkus et al. [19] found significant deficits in processing speed and attention in older adults who were exposed to trauma in childhood, suggesting that the effects last for many years after the trauma exposure. Like Velikonja et al. [20], we also found cognitive deficits in CHR for psychosis, indicating that trauma may heighten vulnerability in already at-risk individuals.

Particularly prominent were memory deficits, both working and long term. Trauma exposed adults show significant impairments in spatial working memory, as noted by Majer et al. [21], and these deficits appear to be associated with hippocampal dysfunction. These findings were further corroborated by Enlow et al. [22], who showed a reduction in IQ scores among children exposed to early interpersonal trauma. The findings are consistent with the idea that childhood trauma interferes with the development of brain areas needed for memory and learning, including the hippocampus and prefrontal cortex.

It also had a significant effect on emotional functioning. Huh et al. [24] and Motsan et al. [23] showed that trauma exposure impairs emotion recognition and regulation and that this is mediated by maladaptive coping strategies. However, these studies show that trauma-exposed individuals have difficulty regulating their emotional responses, making them more vulnerable to depression and anxiety. Studies like Marusak et al. [25] explored the neurobiological underpinnings of these findings, finding heightened amygdala activity and disrupted connectivity between the amygdala and pregenual anterior cingulate cortex (pgACC) in trauma-exposed children. The observed difficulties in emotional regulation and processing probably result from these neural alterations.

The potential reversibility of trauma-induced deficits was demonstrated by intervention studies. Neurofeedback training (NFT) to reduce PTSD symptoms and improve executive functioning in children who experienced developmental trauma was demonstrated by Rogel et al. [26]. Nevertheless, the durability of these improvements was variable, indicating that additional intervention or supplementary therapies may be required.

The findings from the data synthesis underscore the profound and multifaceted impact of early childhood trauma on cognitive and emotional development. Subgroup analyses revealed significant deficits across key domains, including processing speed, attention, working memory, emotion regulation, and executive function. The most pronounced impairments were observed in attention (SMD = 2.37, 95% CI: [5.75, 10.50]) and working memory (SMD = 3.55, 95% CI: [2.18, 9.28]), highlighting the susceptibility of these cognitive functions to trauma-related disruptions. Similarly, emotional regulation difficulties (SMD = 1.25, 95% CI: [1.12, 3.62]) and executive function impairments (SMD = 1.61, 95% CI: [0.06, 3.28]) were evident, aligning with previous research on the role of trauma in emotional dysregulation and compromised higher-order cognition.

Although these results provide compelling evidence for the adverse effects of trauma, significant heterogeneity was observed across all domains (I^2^ ranging from 77% to 98%), reflecting variability in the studies’ methodologies, populations, and assessment tools. This heterogeneity highlights the complexity of trauma’s impact, influenced by factors such as trauma type, duration, timing, and individual resilience. Despite this variability, the overall pooled effect size (SMD = 1.57, 95% CI: [−0.12, 3.26]) reinforces the pervasive nature of trauma-related impairments. These findings underscore the urgent need for targeted interventions that address domain-specific vulnerabilities and mitigate the long-term consequences of childhood trauma.

## Limitations

Several limitations are to be acknowledged, while the findings of this review are robust. First, the study designs, sample populations, and outcome measures were all quite heterogeneous, leading to variability in effect sizes and the comparability of results. For instance, some studies had targeted childhood populations, others adults, and the outcomes measured varied from specific cognitive domains to broader emotional functioning. Meanwhile, the differences in age at trauma exposure and the heterogeneity of trauma types should be analyzed in subcategories with emphasis. We believe that the experience of suffering in early years can affect a person’s entire life. In future research, we will do our best to find a large body of literature that can be included in the analysis, subdivide age and conduct subgroup meta-analysis, so as to obtain the influence curve of early painful experiences with increasing age.

Second, in most studies, reliance on retrospective self-reports of trauma exposure is prone to recall bias. Retrospective assessments may miss or underestimate the severity of trauma and thus may lead to bias in results. Causal inferences would be strengthened by prospective longitudinal studies with objective measures of trauma exposure.

Third, although most studies have large sample sizes, some intervention studies have relatively small sample sizes, which may lower statistical power and reduce the reliability of estimates for effect size. These studies offer useful insights, but larger and more varied samples are needed to confirm the findings and increase generalizability. Some individuals, due to their strong endurance, have little impact on their growth and future life from the experiences of early torture and abuse. These individuals should be separately classified into a subgroup for physiological and psychological analysis to obtain results that are helpful for psychotherapy and recovery. In our future research, we will expand the reading of literature and reports and try to find these individuals for analysis as much as possible.

This review finally did not examine systematically cultural and contextual factors. The outcomes and their interpretation may be influenced by cultural and socioeconomic contexts and experiences with trauma, resilience factors, and access to resources. In our future research, we will continuously improve the design, expand the sample size, take into account external factors influencing psychology, such as economic factors and social culture, and study the impact of early childhood trauma as comprehensively as possible.

## Clinical implications

The findings of this review have important implications for clinical practice and public health. The need for early identification and intervention of childhood trauma is clear, first and foremost due to its persistent and multifaceted impact. Trauma exposure and its cognitive and emotional consequences should be routinely screened in pediatric and mental health assessments. Such screenings could aid in the early identification of at-risk individuals and offer timely support.

Second, the findings are consistent with the use of trauma informed care frameworks across health care, education and social services. These frameworks focus on understanding and working with trauma without retraumatizing. For instance, educational interventions that offer cognitive and emotional support to trauma exposed children may buffer against long term academic and psychological problems.

The third is that trauma-induced deficits can be reversed by interventions such as neurofeedback training and cognitive behavioral therapies, and therefore, the integration of evidence-based treatments into clinical practice is important. Interventions should be tailored to the individual’s specific needs, with monitoring to determine whether benefits are sustained.

## Conclusion

This systematic review and meta-analysis suggest that early childhood trauma has pervasive and lasting effects on cognitive, emotional, and neurodevelopmental outcomes. These findings underscore the urgent need for early prevention and intervention strategies to prevent the harmful outcomes of trauma. Trauma is a complex issue that necessitates clinical, educational, and societal interventions. Future research should use longitudinal designs to explore developmental trajectories of trauma-exposed individuals, culturally sensitive methodologies to account for contextual factors, and larger, more diverse samples to enhance generalizability.

## Supplementary Material

Manuscript_Clean.docx

PRISMA_2020_checklist.docx

## Data Availability

The datasets used and/or analyzed during the current study are available from the corresponding author on reasonable request.
